# Gravity-Driven Microfluidic Viscosity Measurement with a Small Capillary Radius and Strong Pinning Effect

**DOI:** 10.3390/mi17050580

**Published:** 2026-05-07

**Authors:** Jian Dong, Bilong Liu, Xuxuan Ai, Qihang Zhang

**Affiliations:** 1 Key Laboratory of E&M, Zhejiang University of Technology, Hangzhou 310023, China; 201806040711@zjut.edu.cn (B.L.);; 2 State Key Lab of Transducer Technology, Shanghai Institute of Microsystem and Information Technology, Chinese Academy of Sciences, Shanghai 200031, China

**Keywords:** microviscometer, capillary pinning, image-based viscosity measurement, microfluidics

## Abstract

In this study, we introduce a novel method for microscale viscosity measurement that eliminates the need for direct contact angle determination. By utilizing a capillary with a sufficiently small radius (*R* < 0.2 mm), the sharp outlet edge pins the three-phase contact line, stabilizing the apparent contact angle near 90° and nullifying the capillary pressure term. The rheological parameters (*K* and *n*) of power-law fluids are then calculated directly by analyzing image sequences of a growing pendant droplet to obtain its volume flow rate *Q*. Experiments verify through inversion calculation that the apparent contact angle indeed converges to 90° at a small capillary radius. The proposed method is employed to measure 20 wt% and 40 wt% glycerol aqueous solutions (Newtonian fluids) as well as 0.01 wt% and 0.02 wt% xanthan gum aqueous solutions (non-Newtonian fluids). The obtained rheological parameters agree well with reference values within this range, confirming the method’s reliability for these low-viscosity and moderately non-Newtonian fluids. However, measurements on higher concentration fluids (e.g., 0.1 wt% and 0.2 wt% xanthan gum solutions) reveal increased errors, indicating a current limitation in accurately characterizing fluids with high viscosity or pronounced non-Newtonian behavior under gravity-driven flow. This simple technique provides a reliable and low-cost approach for measuring the viscosity of microliter-volume fluids within its characterized operational range.

## 1. Introduction

With the rapid development of biomedical diagnostics, lab-on-a-chip systems, and new material synthesis, there is an urgent demand for the rapid, low-cost, in situ detection of fluid viscosity at micro- to nanoliter volumes [1,2,3,4,5,6,7]. As a key transport property of fluids, small variations in viscosity often carry critical information. For instance, in disease diagnosis, changes in blood or plasma viscosity are closely associated with cardiovascular diseases, diabetes, and other conditions [8,9]. Furthermore, in microfluidic chips, the real-time monitoring of fluid viscosity within channels is essential for cell analysis and drug screening [10,11,12]. Therefore, the development of accurate and convenient microscale viscosity measurement techniques holds important scientific significance and application value.

Among various techniques, the gravity-driven capillary method (e.g., Ubbelohde viscometer) is widely adopted owing to its simple principle and low cost, with its theoretical foundation established on the Hagen–Poiseuille law [13,14]. Nevertheless, when the measurement scale is reduced to the micrometer level, the capillary pressure generated by the outlet meniscus (*P_c_ = 2σ*cos*θ/R*) becomes comparable in magnitude to the gravitational pressure difference (*ρgL*), acting as a non-negligible source of error [15,16]. This raises a fundamental challenge: viscosity calculation is highly dependent on the dynamic contact angle *θ*, whereas the accurate acquisition of *θ* itself remains a difficult task. Firstly, the contact angle *θ* is not an intrinsic material parameter. It is affected by surface roughness, chemical heterogeneity and fluid history, accompanied by obvious hysteresis. Its value dynamically fluctuates within a certain range, making it difficult to determine a single true value [17,18,19]. Such “contact angle uncertainty” directly causes calculation errors in capillary pressure *P_c_*, which is further amplified into considerable systematic deviations in viscosity measurements, forming the core bottleneck restricting the accuracy of microscale viscosity measurement.

To address this issue, existing studies are mainly carried out along two directions. The first is to develop sophisticated imaging technologies and algorithms for real-time fitting of dynamic contact angles [20,21]. The second is to modify capillary surfaces (e.g., superhydrophobic coating) to fix the contact angle *θ* [22]. However, the former complicates the measurement system and falls into a vicious cycle that requires solving another difficult problem for viscosity testing. The latter may introduce new interferences such as surface contamination and coating aging, while sacrificing the universality and convenience of the measurement method. None of the above strategies can fundamentally eliminate the direct dependence on contact angles. Accordingly, there is an urgent research gap for a microscale viscosity measurement method that avoids contact angle measurement and does not introduce additional complexity. Therefore, this study proposes that for capillaries with an extremely small inner diameter (*R* < 0.2 mm), the strong geometric constraint at the sharp outlet edge induces an obvious contact line pinning effect [23,24]. Under gravity-driven flow, this effect stabilizes the apparent contact angle *θ* near 90°, thereby driving the capillary pressure term *P_c_* to approach zero. This physical mechanism greatly simplifies the flow rate formula and fundamentally removes the requirement for direct contact angle measurement.

Accordingly, in this study, we first establish a steady-state flow model under the competition between gravity driving and capillary pressure in a vertical capillary and derive a generalized flow rate formula. Systematic experiments further demonstrate that the apparent contact angle obtained through inversion indeed approaches 90° as the capillary radius decreases, verifying the dominant role of the pinning effect. Finally, by fixing the contact angle at 90°, we develop a novel viscosity measurement method based on image sequences of growing pendant droplets. This method allows for the direct calculation of viscosity only by measuring the droplet volume growth rate (flow rate) and using known physical properties (density, surface tension). Tests on glycerol aqueous solutions with various concentrations show that the measured results agree well with the standard values, validating the reliability of the proposed method.

The research presented in this paper not only provides a novel, low-cost strategy for microscale viscosity measurement with a simple principle and convenient method of operation but also elucidates how to actively control flow boundary conditions by exploiting the strong pinning effect at the microscale, offering new insights for the design and analysis of microfluidic systems.

## 2. Theory and Methods

### 2.1. Gravity-Driven Capillary Flow Under Pendant Drop Boundary Conditions

**Figure 1:**
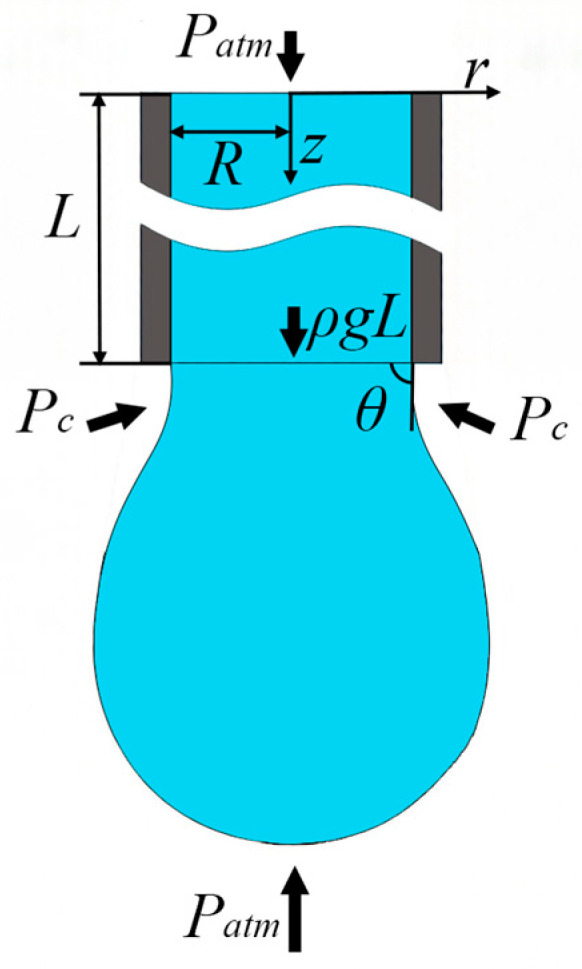
Gravity-driven capillary flow model based on pendant drop boundary conditions.

As shown in Figure 1, a vertical circular capillary with radius *R* and length *L* is considered, which is placed vertically with the positive *z*-axis pointing downward, consistent with the direction of gravitational acceleration *g*, and filled with an incompressible power-law fluid. The model is established under the following assumptions:

1. The system has a fully developed, steady, and axisymmetric laminar flow, with a Reynolds number is well below the critical Reynolds number for laminar flow. Viscous forces dominate completely, and the effect of inertial forces can be neglected.

2. The fluid is an incompressible power-law fluid, and its constitutive relation is given by:

(1) $$\tau_{rz}=K{\left|\frac{du}{dr}\right|}^{n-1}\frac{du}{dr}$$

where $\tau_{rz}$ is the shear stress, u=u(r) is the axial velocity, *K* is the consistency coefficient (Pa·s^n^), and *n* is the power-law index (dimensionless).

When *n* = 1, the fluid behavior reduces to that of a Newtonian fluid, and *K* corresponds to the dynamic viscosity *μ*.

When *n* < 1, the fluid exhibits shear-thinning behavior (pseudoplastic fluid).

When *n* > 1, the fluid exhibits shear-thickening behavior (dilatant fluid).

3. The fluid properties are constant, including the density *ρ*, consistency coefficient *K*, power-law index *n* and surface tension *σ*.

4. The capillary wall exhibits uniform wettability, with a static contact angle *θ* (0 < *θ* ≤ 90°, wetting condition) between the fluid and the wall.

For steady, incompressible, and axisymmetric axial laminar flow, the axial component of the Navier–Stokes equation in the cylindrical coordinate system is simplified as:

(2) $$0=-\frac{dP}{dz}+\frac{1}{r}\frac{d}{dr}\left(r\tau_{rz}\right)+\rho g$$

where P is the driving pressure.

The pressure at the capillary inlet (*z* = 0) is the atmospheric pressure *P_atm_*. A meniscus exists at the outlet (*z = L*), and the capillary pressure is given by *P_c_* = 2*σ*cos*θ*/*R*. Consequently, the outlet pressure satisfies *P(L) = P_atm_ + P_c_*.

Therefore, the total pressure difference along the capillary and the approximately constant pressure gradient are expressed as:

(3) $$\frac{dP}{dz}\approx \frac{P\left(L\right)-P\left(0\right)}{L}=\frac{P_{c}}{L}=\frac{2\sigma \cos\theta }{RL}$$

Capillary pressure *P_c_* appears in the momentum equation as a pressure gradient term *P_c_ /L* along the capillary length that resists gravity-driven flow. This method, which converts the local boundary pressure condition into a net pressure driving force (gradient) acting on the control volume (the entire fluid column), is consistent with the strategy adopted by Lunati [25]. They introduced the capillary pressure term to correct the classical Poiseuille flow in the study of liquid slug motion within gravity-driven capillaries.

Substitute Equation (3) and Equation (1) into Equation (2). Considering that d*u*/d*r* < 0 in the capillary, the term is expressed as (−du/dr)n) to standardize the sign and rearrange to obtain:

(4) $$\frac{K}{r}\frac{d}{dr}\left[r{\left(-\frac{du}{dr}\right)}^{n}\right]=\frac{P_{c}}{L}-\rho g$$

We define the net driving pressure gradient *G* as the difference between the gravity term and the capillary pressure gradient term:

(5) $$G=\rho g-\frac{P_{c}}{L}=\rho g-\frac{2\sigma \cos\theta }{RL}$$

Equation (4) can then be rewritten in its standard form:

(6) $$\frac{1}{r}\frac{d}{dr}\left[r{\left(-\frac{du}{dr}\right)}^{n}\right]=-\frac{G}{K}$$

By integrating Equation (5) twice and applying the boundary conditions, we determine the following:

1. The shear rate d*u*/d*r* is finite at the capillary axis (*r* = 0).

2. The no-slip condition *u*(*R*) = 0 is satisfied at the tube wall (*r* = *R*).

The axial velocity profile of the power-law fluid in the capillary is derived as follows:

(7) $$u\left(r\right)=\frac{n}{n+1}{\left(\frac{G}{2K}\right)}^{1/n}\left[R^{\left(n+1\right)/n}-r^{\left(n+1\right)/n}\right]$$

Integrating the velocity profile across the cross-sectional area gives the steady-state volumetric flow rate:

(8) $$Q=\int_{0}^{R}2\pi ru\left(r\right)dr=\frac{\pi n}{3n+1}{\left(\frac{G}{2K}\right)}^{1/n}R^{\left(3n+1\right)/n}$$

Substituting *G* from Equation (5) and the expression for capillary pressure *P_c_* yields the final steady-state flow rate formula applicable to power-law fluids:

(9) $$Q=\frac{\pi n}{3n+1}{\left(\frac{1}{2K}\right)}^{1/n}{\left(\rho g-\frac{2\sigma \cos\theta }{RL}\right)}^{1/n}R^{\left(3n+1\right)/n}$$

When *n* = 1, the flow rate formula for power-law fluids reduces to that for Newtonian fluids. This is formally symmetric to the governing equation for capillary rise presented by Fries [26]. Under steady-state conditions, the latter reduces to Jurin’s law, whereas the formula in the present work describes the complementary case of gravity-driven downward flow with capillary resistance at the outlet.

Equation (8) clearly reveals two competing mechanisms: the gravitational term *ρ*g drives the fluid downward, while the capillary pressure gradient term *2σcosθ/(RL)* acts upward and resists the flow. This term is inversely proportional to the capillary radius *R*, and thus its effect is significant at the microscale and cannot be neglected.

### 2.2. Strong Pinning Effect at Small Capillary Radius

When a sharp edge exists at the capillary outlet, the contact line becomes pinned at the edge. According to the Gibbs inequality criterion [27,28], the condition for pinning is

(10) $$\theta_{c}<\theta <\left(180^{{}^{\circ}}-\alpha \right)+\theta_{c}$$

where *θ_c_* represents the intrinsic contact angle, *θ* represents the apparent contact angle under the pinned state, and α represents the edge angle. For a typical, unmodified vertical capillary outlet, the edge can be regarded as a perpendicular corner, i.e., α = 90°. Substituting this into the above equation, the range of the pinning angle is simplified to

(11) $$\theta_{c}<\theta <90^{{}^{\circ}}+\theta_{c}$$

From the capillary pressure equation, it can be seen that when *θ* = 90°, cos*θ* = 0, and the capillary pressure becomes zero. Under the pinned state, the system tends to select an equilibrium contact angle that minimizes the total interfacial energy. At *θ* = 90°, the meniscus is planar with zero curvature, corresponding to the lowest interfacial deformation energy. Therefore, when the intrinsic contact angle *θ_c_* < 90°, driven by both geometric constraints (sharp-edge pinning) and the principle of energy minimization, the apparent contact angle exhibits a strong tendency to stabilize near 90°, such that the capillary pressure term vanishes and the system reaches a mechanically simple equilibrium state.

When the capillary radius *R* is sufficiently small, the curvature at the outlet edge becomes extremely large, and the geometric constraint effect is significantly enhanced. This forces the liquid–gas interface to strictly comply with the geometric boundary at the sharp edge, thereby greatly limiting the deviation of the contact angle from 90°. On the basis of experimental observations, this study identifies an empirical critical condition for capillary diameter (*R* < 0.2 mm), at which the strong pinning effect stabilizes the apparent contact angle around 90°.

In accordance with the Gibbs inequality, the stability of contact line pinning is determined by the energy state of the solid–liquid–gas three-phase system at sharp edges. For capillaries with a large radius R, the meniscus has a large radius of curvature. The detachment of the contact line from the pinning position, which induces the deviation of *θ* from 90°, leads to a relatively small increase in interfacial energy, resulting in weak pinning. When *R* is reduced to a scale comparable to or smaller than the capillary length *λ_c_ =* (*σ*/*ρg*)^1/2^, the geometric confinement of the sharp edge becomes dominant. Any displacement of the contact line must overcome a considerable interfacial energy barrier, consequently generating strong contact line pinning.

Taking deionized water as an example, its capillary length is *λ_c_ =* 2.7 mm. The threshold radius measured in this study is *Rth ≈* 0.2 mm, yielding *Rth*/*λ_c_ ≈* 0.074, which is far smaller than the capillary length. This reveals that the strong pinning effect is a typical characteristic of microscale flow (*R* ≪ *λ_c_*). Future research will focus on developing a more comprehensive theoretical model that incorporates dynamic contact angle hysteresis, fluid constitutive equations and surface heterogeneity, so as to quantitatively predict the critical pinning radius for diverse fluid–solid surface combinations.

Based on the above theoretical framework, systematic experimental observations in this study confirm that for the specific stainless-steel capillaries and fluid systems adopted herein, the pinning effect is sufficiently strong to stabilize the apparent contact angle *θ* near 90° when the capillary radius satisfies *R* < 0.2 mm. Accordingly, small-diameter capillaries meeting this criterion serve as a critical experimental prerequisite to achieve stable contact line pinning and thereby neglect capillary pressure. Moreover, this condition is independent of the fluid constitutive behavior, whether Newtonian or non-Newtonian. For power-law fluids, the relation cos*θ ≈* 0 can also be valid under the strong pinning condition of small capillary radii, which drives the capillary pressure *P_c_* to approach zero and greatly simplifies the measurement. On this basis, the flow rate Equation (9) can be simplified as:

(12) $$Q=\frac{\pi n}{3n+1}{\left(\frac{\rho g}{2K}\right)}^{1/n}R^{\left(3n+1\right)/n}$$

This equation provides a theoretical foundation for the viscosity measurement of power-law fluids (rheological parameters *K* and *n*) based on the pinning effect while eliminating the need for contact angle determination. Using multiple sets of experimentally measured data pairs (*R_i_*,*Q_i_*), the rheological parameters *K* and *n* of the fluid can be inversely derived via nonlinear fitting.

### 2.3. Theoretical Model of Axisymmetric Pendant Droplets

The growth dynamics of a droplet are jointly determined by inertial force, viscous force, gravity, and interfacial tension. Within the flow rate range concerned in this study, both the Bond number (Bo = *ρgR*^2^/*γ*) and Reynolds number (Re = *ρvR*/*μ*) of the droplet are small, indicating that interfacial tension and gravity are the dominant factors governing the droplet shape, while the contributions of inertial and viscous forces to the shape can be neglected. Meanwhile, the capillary tip is fabricated with high precision, and the force field is highly symmetric about its central axis. Accordingly, the droplet exhibits perfect rotational symmetry at this stage, with its outer surface forming a surface of revolution around the capillary axis. In this case, the instantaneous profile of the droplet is governed by the Laplace equation of static equilibrium, and thus can be well described and fitted by the Bashforth–Adams (B–A) theoretical model.

**Figure 2:**
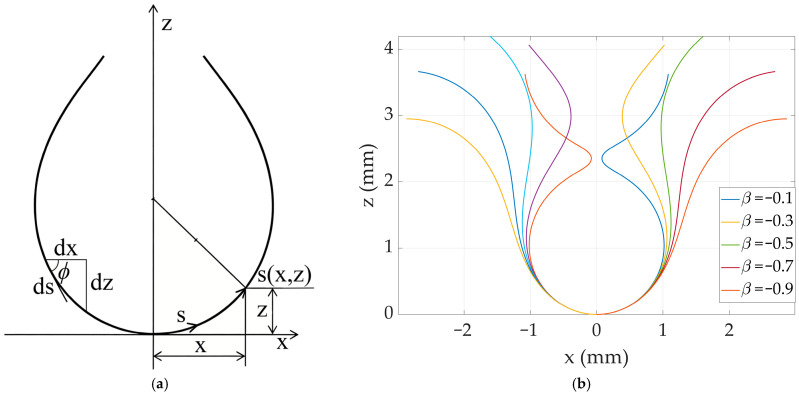
Bashforth-Adams (B-A) pendant drop model and family of droplet profile curves. (**a**) Definition of Geometric Parameters for Axisymmetric Pendant Droplets; (**b**) Family of theoretical profile curves generated by different shape factors *β* at a fixed vertex radius of curvature (b = 1).

As shown in Figure 2a, *o* is the coordinate origin, x and z are the horizontal and vertical coordinates of point *s* on the droplet surface, and *φ* is the tangential angle at point *S*.

Bashforth and Adams derived the Bashforth–Adams equation (B–A equation) based on the Young–Laplace formula [29,30], which describes the axisymmetric droplet at static equilibrium, as shown in Equation (12):

(13) $$\frac{z^{\prime \prime }}{{\left(1+{z^{\prime }}^{2}\right)}^{\frac{3}{2}}}+\frac{z^{\prime }}{x{\left(1+{z^{\prime }}^{2}\right)}^{\frac{1}{2}}}=\frac{2}{b}+\frac{(\rho_{v}-\rho_{1})gz}{\gamma_{\mathrm{lv}}}$$

where ρ_l_ and ρ_v_ are the densities of the liquid and air, respectively, γ_lv_ is the liquid–gas interfacial tension, g is the gravitational acceleration, and b is the radius of curvature of the droplet.

Noumann et al. [31,32,33] modified the B–A equation into a first-order arc differential equation (ARC–B–A equation), as follows:

(14) $$\frac{d\phi }{ds}+\frac{\sin\phi }{x}=\frac{2}{b}+\frac{\beta z}{b^{2}}$$

where *s* is the arc length measured from the origin *o* along the arc differential. The shape factor *β* is defined as $\beta =\left[\left(\rho_{v}-\rho_{l}\right)gb^{2}\right]/\gamma_{lv}$.

As shown in Figure 2a, the following differential identities are derived from the geometric definition parameterized by arc length:

(15) $$\frac{dx}{ds}=\cos\phi ,\frac{dz}{ds}=\sin\phi$$

On the x*-o-z* plane, a suitable representation of the droplet profile is in parametric form:

(16) x=x(s),z=z(s),ϕ=ϕ(s)

In this representation, both *x* and *z* are single-valued functions of s. The boundary conditions for the droplet profile are as follows:

(17) x(0)=z(0)=ϕ(0)=0

Therefore, for given values of *b* and *β*, the droplet profile can be obtained by simultaneous integration. Figure 2b shows the profiles of droplets with *b* = 1 and *β* ranging from −0.9 to −0.1. These profiles form a family of bundle-centered curves that constitute the admissible profile space for axisymmetric droplets.

### 2.4. Experimental Image Profile Fitting and Volume Calculation

The above B-A model and its parameterized family of curves provide a theoretical framework for inversely deriving the physical state of a droplet from its geometric profile. To apply this framework to experimental measurements, the core step is to find the curve from this family that best matches the real-time captured droplet profile, so as to determine the actual values of the parameters (*b*, *β*) and further calculate the volume.

**Figure 3:**
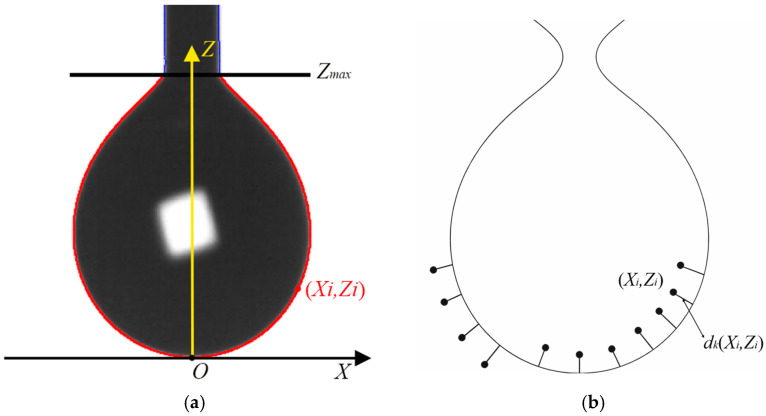
Experimental identification of droplet contours and error evaluation of B-A model fitting. (**a**) Droplet contours extracted from high-speed image sequences. The gas–liquid interface (red), capillary outer contour (blue), and coordinate axes (X-axis in black, Z-axis in yellow) are extracted from the processed image. (**b**) Schematic of the normal distance error between sampling points and the optimal B-A fitting curve.

For each frame of droplet images captured at high speed, grayscale conversion, contrast enhancement, and median filtering are first performed to suppress noise. Subsequently, edge detection algorithms such as the Canny operator are used to automatically identify the coordinate set {(*X_i_*, *Z_i_*)} of the droplet’s gas–liquid interface profile, where the Z-axis direction is opposite to gravity, and the origin *O* is located at the lowest point of the droplet, as shown in Figure 3a. The nozzle edge coordinate Z_max_ is input as a known boundary condition to determine the attachment point of the profile.

An error function based on the normal distance is defined to find the B-A curve that best matches the actual profile: the sum of the squared normal distances from all experimental profile sampling points to a given theoretical B-A curve is calculated to quantify the overall deviation between the curve and the experimental data. By minimizing this error function, the optimal parameters (*b*, *β*) can be objectively determined, providing a clear mathematical basis for the inverse calculation of parameters such as surface tension.

As shown in Figure 3b, the error function between the droplet profile sampling points and a B-A curve is given by:

(18) $$E_{k}=1/2\sum_{i=1}^{N}{\left[d_{k}(X_{i},Z_{i})\right]}^{2}$$

It is the normal distance from a point to the polynomial B-A curve mentioned in the above item. The pair (*b*, *β*) corresponding to the minimum error is found to be the optimal fitting parameters. Substituting into Equation (12) yields the uniquely determined B-A profile curve *x*(*z*) or its parametric form *x*(*s*), *z*(*s*). Since the droplet is axisymmetric, its volume *V* can be obtained by integrating the profile curve rotated around the symmetric axis (the *z*-axis):

(19) $$V=\pi {\int_{0}^{Z_{\max}}\left[x(z)\right]}^{2}dz$$

where *Z*_max_ corresponds to the nozzle plane of the droplet. In actual numerical calculations, the fitted B–A curve is discretely sampled, and the Simpson method is then used for numerical integration to obtain the instantaneous droplet volume *V* with high precision.

## 3. Results

Droplets were generated on the experimental platform using syringes equipped with stainless-steel capillaries of varying inner diameters and a fixed length *L* = 50 mm. A high-speed camera was utilized to capture the evolving profiles of growing droplets. In the present experiments, the capillary length (*L* = 50 mm) was selected to satisfy two fundamental hydrodynamic criteria. Firstly, the Reynolds number Re was guaranteed to be far lower than the critical Reynolds number Re_crit_ for laminar flow (Re ≪ Re_crit_). The flow Reynolds number was defined as Re *= 2ρQ/(πμR)*. The classical critical value Re_crit_ = 2000 was adopted in this study. Secondly, the capillary length *L* was required to be much larger than the entrance length *L*_e_ for fully developed flow, expressed as (*L* ≫ *L*_e_ ≈ 0.12 Re*R*). The capillary radius investigated in the experiments ranged from 0.130 mm to 0.375 mm. This interval was determined according to preliminary experimental observations. Capillaries with radii larger than 0.500 mm failed to produce separate, non-coalescent droplets. In contrast, for radii smaller than 0.110 mm, liquid climbed along the outer orifice wall, breaking the axisymmetry essential for stable droplet growth and detachment.

All capillaries were precisely cut to ensure flat end faces, and microscopic inspection confirmed sharp edges free of visible burrs or defects. Prior to testing, each capillary was ultrasonically cleaned with deionized water and acetone, followed by drying for subsequent use. All experiments were conducted in a thermostatted chamber with the ambient temperature maintained at (20.0 ± 0.1) °C.

**Figure 4:**
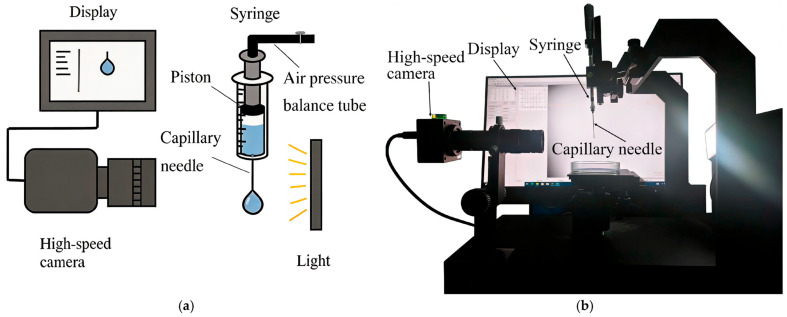
Experimental setup. (**a**) Schematic of the setup; (**b**) Photograph of the setup.

**Figure 5:**
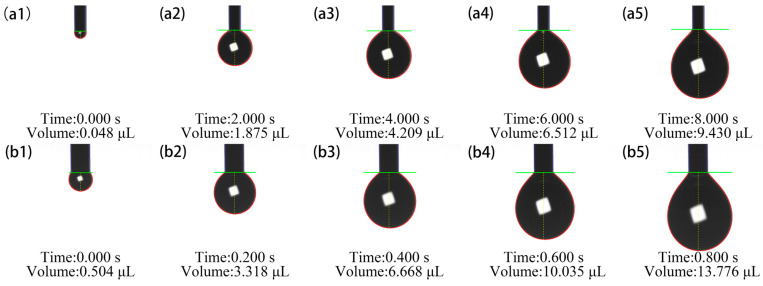
Growth process of pendant droplets under high-speed camera.The gas–liquid interface (red), capillary outer contour (blue), tube orifice baseline (green), symmetry axis (yellow) are extracted from the processed image. (**a1**–**a5**) The capillary radius *R* = 0.130 mm; (**b1**–**b5**) The capillary radius *R* = 0.255 mm.

During the experiments, it was observed that droplets remained in a metastable state at the capillary orifice due to capillary pinning and contact angle hysteresis, and could not flow out spontaneously under gravity alone. To address this issue, the experimental procedure is described as follows: First, an additional initial pressure was briefly applied by rotating a differential micrometer to overcome the initial energy barrier induced by capillary pinning and contact angle hysteresis. Once the first droplet detached and continuous flow was established, the system transitioned to a steady flow dominated by the self-weight of the fluid; thereafter, the initial pressure was removed, and the flow could still be stably sustained. Next, the pressure balance tube connected to the syringe piston was opened to equalize the upstream pressure to the atmospheric condition. Specifically, this tube directly communicated the air cavity behind the syringe piston with the ambient atmosphere, ensuring that the static pressure at the capillary inlet (*z* = 0) remained equal to the atmospheric pressure regardless of the liquid level inside the syringe, while the minor air flow resistance in the connecting tube was neglected. Thus, after steady flow was fully developed, the net driving pressure difference originated solely from the difference between the gravitational head induced by the liquid column self-weight and the capillary pressure at the outlet meniscus, which approached zero under strong pinning effects. Furthermore, given that the syringe radius was considerably larger than the capillary radius, the variation in the liquid level within the syringe was negligible during short-duration droplet generation. Subsequently, high-speed image acquisition was initiated after the flow rate stabilized and the syringe liquid level approached the 0 mm scale. For consistency in data collection, the first frame was defined as the moment when the previous droplet completely exited the field of view, and the final frame corresponded to the obvious necking before droplet breakup, thereby recording the entire droplet growth cycle. The experimental setup for the above procedure is presented in Figure 4. As illustrative examples, taking deionized water droplets generated with capillary radii of 0.130 mm and 0.255 mm, representative snapshots of droplet evolution and corresponding volume calculation results are illustrated in Figure 5.

To ensure data reliability, five independent repeated experiments were conducted for each defined experimental condition in this study, namely each combination of fluid sample and capillary radius. Each experiment covered the complete procedures of droplet generation, image acquisition, and data processing. The subsequent reported data, including the average flow rate *Q*, inverted contact angle *θ*, and rheological parameters *K* and *n*, were all calculated as the arithmetic mean of results obtained from repeated tests under identical experimental conditions.

### 3.1. Verification of Droplet Behavior and Pinning Effect

**Figure 6:**
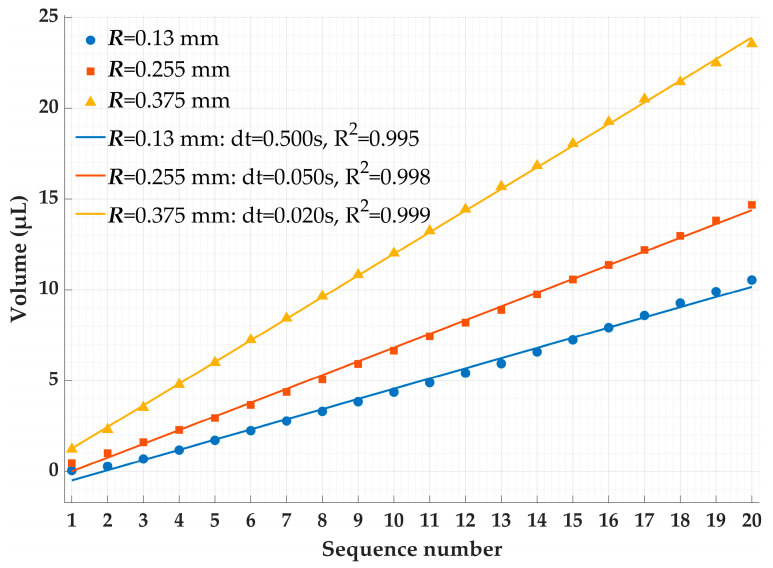
Curve of droplet volume versus frame sequence.

To compare the volume evolution of deionized water droplets at different capillary radii, the time axis was unified using frame numbers. A total of twenty equally spaced frames were extracted from each high-speed image sequence. Afterwards, the droplet volume in each frame was calculated via a numerical algorithm. Representative results were selected to plot the variation in droplet volume versus frame sequence, as presented in Figure 6.

As shown in Figure 6, during droplet growth, the instantaneous growth rate (i.e., the instantaneous flow rate) is not absolutely constant. For larger capillary radii, small fluctuations in the growth rate that deviate from the ideal linear behavior are clearly visible. However, from the perspective of the overall trend and engineering applications, the amplitude of these local fluctuations is very small relative to the average growth rate. The droplet approximately exhibits the typical mode of pinned growth, in which the three-phase contact line (gas–liquid–solid) is firmly pinned at the nozzle edge. The growth of the droplet is fully reflected in the linear accumulation of volume, while the contact radius remains unchanged. This behavior conforms to the general fluid physics of capillary emitters under low-flow conditions.

Meanwhile, the regression model’s coefficient of determination (R^2^) is higher than 0.98 for all experimental conditions. This statistically supports the rationale of using linear regression results to calculate the average volumetric flow rate. Specifically, the slope of the regression line can be used as the best unbiased estimate of the average volumetric flow rate during the entire droplet growth process, thereby effectively suppressing measurement errors caused by instantaneous fluctuations and accurately characterizing the overall growth rate of the droplet with a single characteristic parameter.

Furthermore, during the entire steady droplet growth stage, the droplet volume increases highly linearly with time, demonstrating a constant volumetric flow rate Q. According to theoretical derivation, under the strong pinning condition for small-diameter capillaries *θ ≈* 90°, namely capillary pressure *P_c_ ≈* 0, the steady value of *Q* in Equation (12) directly corresponds to a constant net driving pressure gradient *G = ρg*. The observed constant flow rate is strictly self-consistent with the assumption that the driving force solely arises from constant gravity. This provides strong indirect experimental evidence that the inlet pressure at the capillary orifice stabilizes at atmospheric pressure after the removal of the starting pressure, and that the subsequent flow is purely gravity-driven.

**Figure 7:**
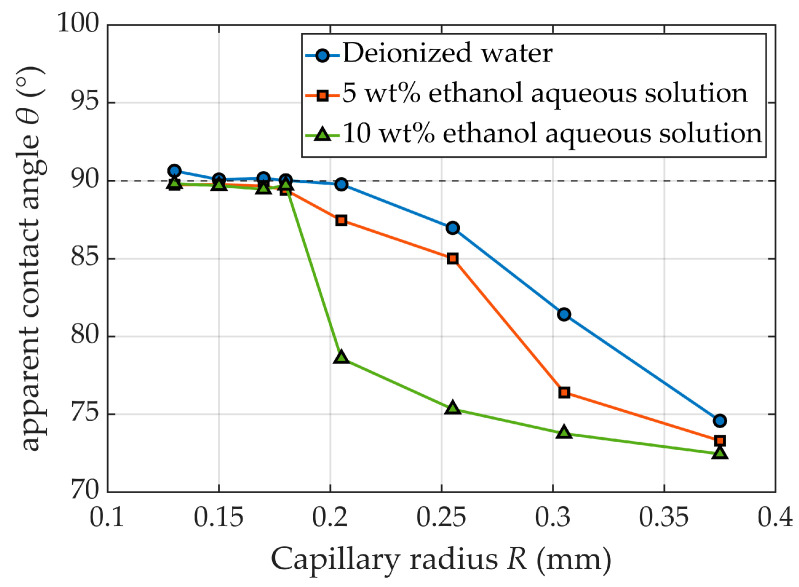
Plot of apparent contact angle versus capillary radii.

To verify the stability and universality of the pinning effect in small-diameter capillaries, deionized water, a 5 wt% ethanol solution, and a 10 wt% ethanol solution were selected as test samples, with their surface tension decreasing sequentially and viscosity increasing sequentially. All samples are Newtonian fluids with *n* = 1. The average flow rate corresponding to each capillary radius and the standard physical properties of the samples were substituted into Equation (8) to calculate the apparent contact angle under various conditions. For each solution, the arithmetic mean of the apparent contact angles within the small-diameter range (*R* < 0.2 mm) was further calculated. All results were summarized in Table 1, and the corresponding variation curve was presented in Figure 7.

The figure above presents the variation in the apparent contact angle inversely calculated from the flow rate formula as a function of capillary radius R. When the capillary radius is sufficiently small (*R* < 0.2 mm), the arithmetic mean contact angles of the three fluids all stabilize near 90° and exhibit no obvious variation with fluid type or flow rate.

To evaluate the influence of these minor residual deviations on the core assumption of the proposed measurement method (*P_c_* ≈ 0), a quantitative analysis was conducted based on the data in Table 1, taking the condition with a relatively large contact angle deviation as a typical example (i.e., deionized water, R = 0.130 mm). The calculated absolute capillary pressure is |*P_c_|* ≈ 12.4 Pa. In the present experimental configuration, the gravity-driven pressure difference generated by a water column with a length (L = 50 mm) is *ρgL* ≈ 490 Pa, with their ratio approximately equal to 2.5%. This result clearly indicates that even considering the maximum residual deviation of the contact angle, the capillary pressure term is far smaller than the gravity term and can be reasonably neglected as a secondary factor. From a statistical perspective, this finding further verifies that simplifying the theoretical model by adopting *θ =* 90° (*P_c_* = 0) under strong pinning conditions is a reasonable, robust, and error-controllable physical approximation.

This phenomenon confirms that under strong microscale confinement, the pinning effect of the sharp nozzle edge on the contact line becomes dominant, suppressing the influence of the intrinsic fluid properties on the contact angle, thereby creating an approximately uniform interface condition governed by geometry. We can therefore draw the key conclusion that within a specific small capillary radius range, the geometric pinning effect enables the dynamic contact angles of different fluids to converge to a stable value (~90°). This effectively establishes and standardizes a unified and repeatable flow boundary condition for our subsequent viscosity measurements.

### 3.2. Fluid Viscosity Measurement Results

To systematically verify the accuracy and universality of the microscale viscosity measurement method proposed in this study for various fluids, the apparent contact angle was set to *θ* = 90°. Five groups of small-capillary experiments were carried out on glycerol aqueous solutions (20 wt% and 40 wt%) and xanthan gum aqueous solutions (0.01 wt% and 0.02 wt%) to obtain the average flow rate *Q*. Nonlinear least-squares regression was adopted to directly fit the experimental data. With the goal of minimizing the sum of squared residuals between predicted and measured flow rates, a bounded nonlinear optimization algorithm (e.g., the fmincon function in MATLAB R2023b, The MathWorks Inc., Natick, MA, USA) was applied to search for optimal solutions in the parameter space. Thereby, the rheological parameters *K* and *n* that enable the optimal matching between theoretical curves and experimental data points were determined as the measured values of the proposed method.

**Figure 8:**
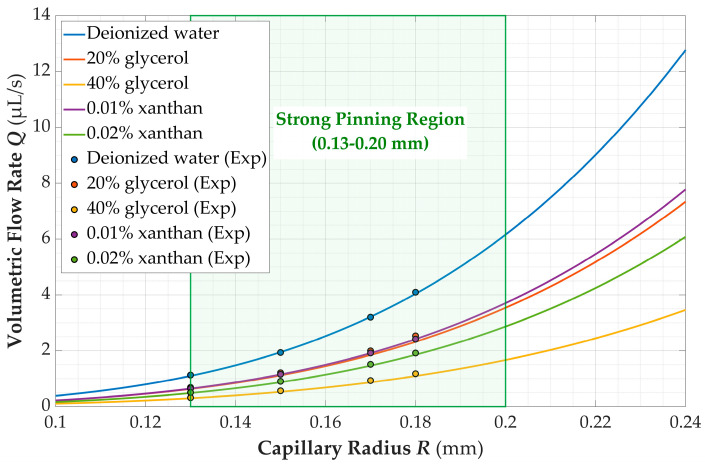
Theoretical prediction and experimental measurement of flow rate versus capillary radius for power-law fluids under strong pinning conditions.

The obtained rheological parameters were compared with two reference benchmarks: the calculated results from the authoritative empirical formula proposed by Cheng [34], and the experimental data reported by Xu [35]. The comparison results were summarized in Table 2 and Table 3. To validate the theoretical model and intuitively demonstrate its predictive characteristics, experimental measurements were compared with theoretical calculations. Based on the experimentally fitted rheological parameters, theoretical flow rate–capillary radius curves were calculated for each fluid. These curves were plotted together with the corresponding experimental data points in linear coordinates, forming the *Q*-*R* relationship illustrated in Figure 8.

## 4. Discussion

Based on theoretical derivation and experimental validation, this study proposes a novel microscale viscosity measurement method relying on the contact line pinning effect at the capillary outlet. The core innovation of this method lies in that, when capillaries with a small inner radius (*R* < 0.2 mm) are adopted, the strong geometric confinement of the sharp outlet edge stabilizes the apparent contact angle near 90°. This condition drives the capillary pressure term to approach zero and fundamentally eliminates the requirement for direct measurement of the dynamic contact angle. By inversely analyzing the flow rates obtained under various capillary radii, the experimental results clearly confirm that the contact angle converges to 90° as the capillary size decreases, which provides direct experimental evidence for the underlying physical mechanism.

Nevertheless, a slight residual deviation in the contact angle still remains. Quantitative analysis indicates that the corresponding relative deviation of the driving pressure introduced by this effect is approximately 2.5%. The influence mechanism of this systematic error on the final rheological parameter measurement varies with fluid type. For Newtonian fluids (e.g., glycerol solutions), the constitutive equation represents a special case of the power-law model at *n* = 1. The fitting algorithm starts with an initial value of *n* = 1, and the final optimized result remains highly close to unity. This indicates that near the optimal solution, the effective dimension of the parameter space degenerates approximately to a single variable dominated by the consistency coefficient *K*. Accordingly, the error propagation originating from the residual contact angle deviation exhibits an approximately linear influence on the fitted *K*, without distortion or amplification through absorption or compensation by the additional variable n. This explains why the measured errors of *K* for Newtonian fluids (approximately 0.6–4.9%) are consistent with the theoretically estimated driving pressure deviation (~2.5%) and remain within an acceptable range.

In contrast, non-Newtonian fluids (e.g., xanthan gum aqueous solutions) possess an intrinsic flow behavior index distinctly lower than 1. When the fitting procedure adopts *n* = 1 as the initial guess, the algorithm must search for the optimal solution far from the initial value within a fully coupled two-parameter space of *K* and *n*. Given that capillary pressure satisfies *P_c_*∝1/*R*, the fixed contact angle deviation imposes nonlinear disturbances on experimental data obtained at different capillary radii. To minimize the overall residual error, the optimization algorithm cooperatively adjusts *K* and n to compensate for such disturbances during fitting, which significantly amplifies the systematic error. This strongly deteriorates the fitting accuracy of the rheological index n, which is highly sensitive to the shear rate (e.g., a relative error of 10.2% for the 0.2 wt% xanthan gum solution in Table 3). Furthermore, the deviation in *n* couples with and propagates to the determined *K*. This underlying mechanism fundamentally accounts for the increased measurement uncertainty of the proposed method when applied to non-Newtonian fluids.

In addition, the reliability of the present method relies on the strong pinning effect under tiny capillary diameters, which inevitably sets an inherent limitation for practical applications. As shown in Figure 8, within the narrow radius range adopted in the experiments (0.13–0.20 mm), the theoretical flow rate–radius curves of different fluids can overlap considerably, such as for 10 wt% glycerol solution and 0.01 wt% xanthan gum solution. This brings about ambiguity in parameter inversion and raises fitting errors, which further accounts for the difficulties encountered in non-Newtonian fluid measurements. Moreover, the low shear rate range induced by gravity-driven flow may restrict the comprehensive characterization of the rheological behaviors of certain non-Newtonian fluids.

To summarize, this study proposes a low-cost and convenient measurement solution for Newtonian and weakly non-Newtonian fluids with micro-scale volume and viscosity close to that of water. For high-viscosity or strongly non-Newtonian fluids, the measurement accuracy is currently limited by the error amplification effect and the applicable range of capillary diameters. Future improvements can focus on expanding the shear rate range through active driving (e.g., micro-pumps) and developing in-situ contact angle calibration technology to correct systematic errors, thereby extending the advantages of this method to a broader range of complex fluid systems.

## Figures and Tables

**Table 1:** Average flow rate and contact angle of different solutions at various capillary radii.

Solutions	Capillary Radius (mm)	Average Flow Rate (μL/s)	Apparent Contact Angle (°)	Average Contact Angle at Small Capillary Radii (°)
Deionized water	0.130	1.117	90.64	90.22
	0.150	1.936	90.08	
	0.170	3.201	90.16	
	0.180	4.007	90.03	
	0.205	6.697	89.77	\
	0.255	15.135	86.97	
	0.305	28.212	81.42	
	0.375	59.547	74.57	
5 wt% ethanol solution	0.130	0.923	89.75	89.64
	0.150	1.638	89.75	
	0.170	2.698	89.67	
	0.180	3.373	89.41	
	0.205	5.448	87.45	\
	0.255	12.622	85.02	
	0.305	22.834	76.40	
	0.375	52.253	73.30	
10 wt% ethanol solution	0.130	0.810	89.81	89.66
	0.150	1.433	89.68	
	0.170	2.353	89.45	
	0.180	2.976	89.71	
	0.205	3.953	78.57	\
	0.255	9.415	75.33	
	0.305	19.684	73.76	
	0.375	46.388	72.44	

**Table 2:** Measured and reference values of rheological parameters for glycerol aqueous solutions of the same concentration at small capillary radii.

Glycerol Mass Fraction (*w*/*w*)	Capillary Radius (mm)	Average Flow Rate (μL/s)	Measured Value of K (mPa·s^n^)	Reference Value of K (mPa·s^n^)	Relative Error of K (%)	Measured Value of n	Reference Value of n	Relative Error of n (%)
20%	0.130	0.687	1.73	1.74	0.6	0.987	1.000	1.3
20%	0.150	1.213						
20%	0.170	1.996						
20%	0.180	2.533						
40%	0.130	0.319	3.87	3.69	4.9	0.986	1.000	1.4
40%	0.150	0.565						
40%	0.170	0.934						
40%	0.180	1.178						

**Table 3:** Measured and reference values of rheological parameters for xanthan gum aqueous solutions of the same concentration at small capillary radii.

Xanthan Gum Mass Fraction (*w*/*w*)	Capillary Radius (mm)	Average Flow Rate (μL/s)	Measured Value of *K* (mPa·s^n^)	Reference Value of *K* (mPa·s^n^)	Relative Error of *K* (%)	Measured Value of *n*	Reference Value of *n*	Relative Error of *n* (%)
0.01%	0.130	0.647	2.18	2.40	9.2	0.966	0.940	2.8
0.01%	0.150	1.157						
0.01%	0.170	1.920						
0.01%	0.180	2.416						
0.02%	0.130	0.504	3.68	4.10	10.2	0.914	0.886	3.2
0.02%	0.150	0.905						
0.02%	0.170	1.514						
0.02%	0.180	1.918						

## Data Availability

The original contributions presented in the study are included in the article, further inquiries can be directed to the corresponding author.

## References

[1] Chin C.D., Linder V., Sia S.K. (2007). Lab-on-a-Chip Devices for Global Health: Past Studies and Future Opportunities. Lab Chip.

[2] Puneeth S.B., Kulkarni M.B., Goel S. (2021). Microfluidic Viscometers for Biochemical and Biomedical Applications: A Review. Eng. Res. Express.

[3] Shankar V., Subramanian G., Hudson S.D. (2022). Microfluidic Viscometers for Shear Rheology of Complex Fluids and Biofluids. Biomicrofluidics.

[4] Chen L., Li Z., Wang Y., Wang L., Li T., Sun L., Wang J., Liu C., Li D., Yang S. (2022). Point-of-Care Blood Coagulation Assay Based on Dynamic Monitoring of Blood Viscosity Using Droplet Microfluidics. ACS Sens..

[5] Park H., Park J., Kim D., Kim D., Jhe W., Han J.C., Lee M. (2024). Deep Learning-Assisted 10-μL Single Droplet-Based Viscometry for Human Aqueous Humor. Lab Chip.

[6] Tang S., Li X., Wang W., Liu H., Wang J. (2025). Droplet Impact-Based Microliter Viscometry (DI-μV). Anal. Chem..

[7] Ge Y., Huang X., Zhang B., Song Z., Tang X., Shao S., Guo L., Liang P., Li B. (2025). Measurement of Fluid Viscosity Based on Pressure-Driven Flow Digital-Printed Microfluidics. Analyst.

[8] Lowe G.D.O., Rumley A., Norrie J., Ford I., Shepherd J., Cobbe S., Macfarlane P. (2022). Plasma and Blood Viscosity in the Prediction of Cardiovascular Disease and Mortality in the Scottish Heart Health Extended Cohort Study. J. Am. Heart Assoc..

[9] Bakhtiari Doost S., Musuroi C., Volmer M., Florescu M. (2024). Optoelectronic Microfluidic Device for Point-of-Care Blood Plasma Viscosity Measurement. Lab Chip.

[10] van Duinen V., Trietsch S.J., Joore J., Vulto P., Hankemeier T. (2015). Microfluidics-Based 3D Cell Culture Models: Utility in Novel Drug Discovery and Delivery Research. Curr. Opin. Biotechnol..

[11] Guo T., Zou X., Sundar S., Jia X., Dhong C. (2023). Label-Free, in Situ Monitoring of Viscoelastic Properties of Cellular Monolayers via Elastohydrodynamic Phenomena. Lab Chip.

[12] Asghari M., Duclos Ivetich S., Aslan M.K., Aramesh M., Melkonyan O., Meng Y., Xu R., Colombo M., Weiss T., Balabanov S. (2024). Real-Time Viscoelastic Deformability Cytometry: High-Throughput Mechanical Phenotyping of Liquid and Solid Biopsies. Sci. Adv..

[13] (2024). Standard Test Method for Kinematic Viscosity of Transparent and Opaque Liquids (and Calculation of Dynamic Viscosity).

[14] Sequeira M.C.M., Caetano F.J.P., Fareleira J.M.N.A. (2024). Capillary Viscometry for Routine Measurements of Newtonian Liquids. Int. J. Thermophys..

[15] Yang G., Li B., Shi W., Li W., Pan H., Gu L., Zhang F., Li B., Zhang R., Wang W. (2020). Liquid Seal for Compact Micropiston Actuation at the Capillary Tip. Sci. Adv..

[16] Lei J., Xu Z., Xin F., Lu T. (2021). Dynamics of Capillary Flow in an Undulated Tube. Phys. Fluids.

[17] Athukorallage B., Aulisa E., Iyer R., Zhang L. (2015). Macroscopic Theory for Capillary-Pressure Hysteresis. Langmuir.

[18] Li C.X., Cheng R., Ye X.M. (2021). Influence of Contact Angle Hysteresis and Temperature Sensitivity of Gas-Liquid Interfacial Tension on Droplet Evaporation Dynamics. Acta Phys. Sin..

[19] Xian Z., Du Z., Chen Y., Liu L., You H. (2023). Dynamic Contact Angle Measurement of Hydrophilic Open Microchannels: The Role of Surface Wettability. Phys. Fluids.

[20] Zhang S., Liu Y., Zhang B., Zhang J., Chen H., Zhou X. (2024). Measurement Methods for Droplet Adhesion Characteristics and Micrometer-Scale Quantification of Contact Angle on Superhydrophobic Surfaces: Challenges and Opportunities. Langmuir.

[21] Wang X., Ke P., Du F. (2024). Research on the Dynamic Contact Angle Model for the Droplet Impact Process. Appl. Math. Mech..

[22] Vuckovac M., Backholm M., Timonen J.V.I., Ras R.H.A. (2020). Viscosity-Enhanced Droplet Motion in Sealed Superhydrophobic Capillaries. Sci. Adv..

[23] Ding H., Chen B.Q., Liu H.R., Zhang C.Y., Liu Y., Zhang P. (2015). On the Contact-Line Pinning in Cavity Formation During Solid–Liquid Impact. J. Fluid Mech..

[24] Shan F., Xiao J., Chai Z., Shi B. (2022). Pinning and Depinning of Imbibition Beyond a Sharp Edge: A Lattice Boltzmann Study. Int. J. Multiph. Flow.

[25] Lunati I., Or D. (2009). Gravity-Driven Slug Motion in Capillary Tubes. Phys. Fluids.

[26] Fries N., Dreyer M. (2008). An Analytic Solution of Capillary Rise Restrained by Gravity. J. Colloid Interface Sci..

[27] Gibbs J.W. (1878). On the Equilibrium of Heterogeneous Substances. Trans. Conn. Acad. Arts Sci..

[28] Oliver J.F., Huh C., Mason S.G. (1977). Resistance to Spreading of Liquids by Sharp Edges. J. Colloid Interface Sci..

[29] Eggers J., Dupont T.F. (1994). Drop Formation in a One-Dimensional Approximation of the Navier–Stokes Equation. J. Fluid Mech..

[30] Tang Z.Y., Li J., Guo Y.K., Duan H.L. (2020). Effects of Viscosity and Surface Tension of a Reactive Dye Ink on Droplet Formation. Langmuir.

[31] Zhang X.G. (1999). Dynamics of Growth and Breakup of Viscous Pendant Drops into Air. J. Colloid Interface Sci..

[32] Killion J.D., Garimella S. (2004). Simulation of Pendant Droplets and Falling Films in Horizontal Tube Absorbers. J. Heat Transf..

[33] Che Z.Z., Duan H.L., Zhang X.Y., Zhang T.H. (2011). Numerical Investigation of Upstream Pressure Fluctuation during Growth and Breakup of Pendant Drops. Chem. Eng. Sci..

[34] Cheng N.S. (2008). Formula for the Viscosity of a Glycerol-Water Mixture. Ind. Eng. Chem. Res..

[35] Xu X.S., Zhao W.B., Li M.Y., Lu Y.Z., Wang S.J. (2021). Experimental study on pipe flow transition of XG solution and drag reduction characteristics with different mass fractions of NaCl. J. Exp. Fluid Mech..

